# Cochlear Implant Complications in Children and Adults: Retrospective Analysis of 148 Cases

**DOI:** 10.7759/cureus.20750

**Published:** 2021-12-27

**Authors:** Mohamed Garrada, Meaad K Alsulami, Samia N Almutairi, Shahad M Alessa, Afaf F Alselami, Nujood A Alharbi, Roaya A Alsulami, Reham Y Talbi, Khaled I Al-Nouri

**Affiliations:** 1 Audiovestibular Medicine, Otolaryngology, Head & Neck Surgery, King Abdulaziz University Faculty of Medicine, Jeddah, SAU; 2 Surgery, King Abdulaziz University Faculty of Medicine, Jeddah, SAU; 3 Medicine, King Abdulaziz University Faculty of Medicine, Jeddah, SAU; 4 Otolaryngology, Head & Neck Surgery, King Abdulaziz University Faculty of Medicine, Jeddah, SAU

**Keywords:** sensorineural hearing loss, hearing rehabilitation, deafness, surgical complications, cochlear implants

## Abstract

Objective

This study aimed to establish and discuss the intraoperative and postoperative complications affecting patients who underwent cochlear implant (CI) surgery from the Cochlear Implant Program of King Abdulaziz University Hospital (KAUH), Jeddah, Saudi Arabia.

Methods

A retrospective study was conducted by reviewing the medical records of 148 patients who underwent cochlear implantation at KAUH between 1999 and 2019. Postoperative complications were classified into minor and major complications. Minor complications resolved with minimal or no treatment. Major complications required additional surgery or hospitalization.

Results

Complications occurred in 28 (18.9%) patients. Minor complications occurred in 17 (11.5%) patients, which included otitis media (2%), facial palsy (1.4%), wound infection (1.4%), vertigo (1.4%), intraoperative cerebrospinal fluid (CSF) gusher (1.4%), tinnitus (1.4%), facial stimulation (1.4%), hematoma (0.7%), and chorda tympani nerve injury (0.7%). Major complications occurred in 11 (7.4%) patients. These included flap dehiscence/infection (2%), device failure (1.4%), device migration (1.4%), mastoiditis (1.4%), electrode damage during insertion (0.7%), and misplaced electrodes (0.7%).

Conclusion

This study reported a low rate of surgical complications associated with CI, and most have been managed successfully without further complications. Our results prove that CI is a safe and reliable procedure, with a low complications rate when performed by experienced surgeons.

## Introduction

Hearing loss is the partial or total inability to hear. It is considered one of the most preventable disabilities worldwide. Hearing loss affects one to two infants in every 1,000 live births [1]. The incidence of congenital hearing loss in Saudi Arabia is relatively higher compared to international figures, affecting one to four infants in every 1,000 live births [2].

Cochlear implants (CIs) have proven to be the most suitable tool for hearing rehabilitation in patients with severe sensorineural hearing loss who cannot benefit from traditional hearing aids. It is an implantable electronic hearing device designed to stimulate the nerves in the inner ear to produce auditory sensations in patients with moderate to severe neurological deafness [3]. Cochlear implants are safe and reliable and provide significant benefits to patients such as improving communication skills and quality of life [4].

CI surgeries are considered safe and have been performed successfully in many patients; however, postoperative complications still occur. These complications are related to surgical technique, foreign body implantation, or device failure. Different types of equipment and techniques have been used to improve the surgical results [5].

Surgical complications were classified according to the research criteria of Cohen et al. in most publications [6]. They classified postoperative complications of CIs into major and minor complications. Major complications require additional surgery or hospitalization while minor complications can be managed with only medications [7-9].

In our institute, 148 pediatric and adult patients have undergone cochlear implantation since the start of the Cochlear Implant Program, which consists of a multidisciplinary team of physicians, speech and hearing therapists, psychologists, and social workers. The primary goal of this study was to evaluate the complications encountered in children and adults of various age groups who underwent cochlear implantation in our center to add more data to the current literature.

## Materials and methods

This study was designed as an observational, descriptive study, and data were collected retrospectively from the medical records of the patients. A total of 148 patients who underwent CI surgery at King Abdulaziz University Hospital (KAUH) between 1999 and March 2019 were the main subjects. All patients had follow-ups for at least one-year post-implantation. The ethical committee of our institution approved this study.

The following data were studied: hearing and medical history, age at implantation, radiological images (computerized tomography and magnetic resonance images), intraoperative findings, and postoperative complications.

Cochlear implantation was performed by a standardized multidisciplinary team. From the surgical point of view, all cases were performed through a postauricular incision and double muscle flap for the device and approached through a posterior tympanotomy. Over the years, the incision has become smaller.

Device fixation, until 2010, was performed using non-absorbable sutures, as recommended by most cochlear implant companies. However, since 2010, all devices are fixed, and a bony wall for the receiver/stimulator is drilled and tailored according to the implant shape and the tight subperiosteal pocket. The approach to the cochlea was through cochleostomy until 2014 and onwards. A round window is used when possible. Facial nerve monitoring is conducted as a standard in all cases since 2011. A pressure dressing is used for 24 hours, and an intravenous third-generation antibiotic is prescribed intraoperatively and continued orally for 10 days after the operation.

According to the study criteria of Cohen and Hoffman, surgical complications were classified into major complications, which require surgical intervention or hospital admission, and minor complications, which require outpatient treatment and observation. Complications were also classified according to the time of occurrence into intraoperative, early postoperative (less than three months), or late postoperative (more than three months).

## Results

This study included 148 patients (162 CIs). All CIs were performed consecutively from 1999 to March 2019 in our department by the same surgeon or under the supervision of the same surgeon.

The series comprised 134 unilateral cochlear implants (90.5%) and 14 bilateral cochlear implants (9.5%). A total of 148 implantations were performed in 136 pediatric and 12 adult patients. The mean follow-up duration was five years. The implant brand MED-EL (Innsbruck, Austria) was the most commonly used (56.8%), followed by Cochlear (36.4%; Sydney, Australia), and Advanced Bionics (6.8%; Stäfa, Switzerland) (Table 1). 

**Table 1:** Characterstics of patients (n=148) and devices (n=162) implanted between 1999 and 2019 (n=148)

	No. of patients	Percentage
Gender		
Male	73	49.3
Female	75	50.7
Age at implantation		
≤18 years	136	91.9
>18 years	12	8.1
Min. – Max.	1.0 – 57.0	
Mean ± SD.	6.83 ± 8.69	
Median (IQR)	4.0 (3.0 – 6.0)	
Follow up duration (years)		
Min. – Max.	1.0 – 20.0	
Mean ± SD.	5.68 ± 4.03	
Median (IQR)	5.0 (3.0 – 8.0)	
Type of implantation	No. of patients	Percentage
Unilateral	134	90.5
Bilateral	14	9.5
Site of implantation	No. of devices	Percentage
Right	100	61.7
Left	62	38.3
Type of cochlear implant device	No. of devices	Percentage
Cochlear	59	36.4
MED-EL	92	56.8
Advanced Bionic	11	6.8

Complications occurred in 28 (19%) patients, including minor complications in 17 (11.5%) and major complications in 11 (7.4%) cases (Table 2, Table 3).

**Table 2:** Distribution of the studied cases according to minor complications (n=17) *Cerebrospinal fluid

Minor complications	No. of cases	% of the total
Otitis media	3	2.0
Facial palsy	2	1.4
Wound infection	2	1.4
Vertigo	2	1.4
Intraoperative CSF* gusher	2	1.4
Tinnitus	2	1.4
Facial stimulation	2	1.4
Hematoma	1	0.7
Chorda tympani nerve injury	1	0.7
Total	17	11.5

**Table 3:** Distribution of the studied cases according to major complications (n=11)

Major complications	No. of cases	% of the total
Flap dehiscence/infection	3	2.0
Device failure	2	1.4
Device migration	2	1.4
Mastoiditis	2	1.4
Damaged electrode	1	0.7
Electrode wrong position	1	0.7
Total	11	7.4

Minor complications included delayed otitis media in three patients, facial nerve palsy in two patients, wound infection in two patients, signs of vertigo in two patients, intraoperative cerebrospinal fluid (CSF) gusher in two patients, tinnitus after surgery in two patients, facial stimulation in two patients, hematoma in one patient, and transient injured chorda tympani during drilling in one patient (Table 2).

**Figure 1:**
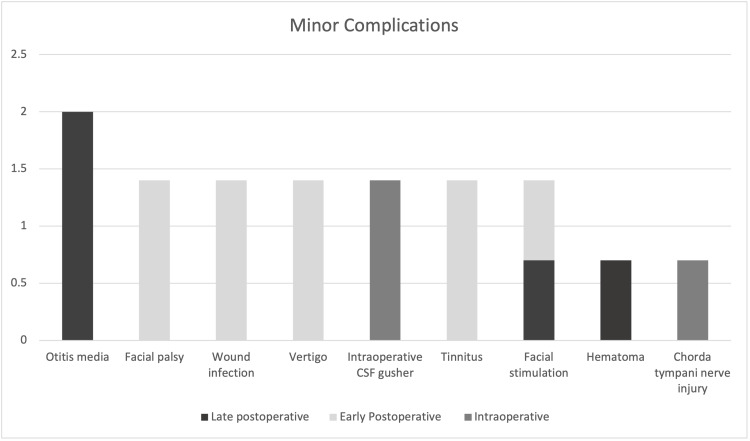
Percentage of complications according to time of appearance (Minor Complications, n=17)

**Figure 2:**
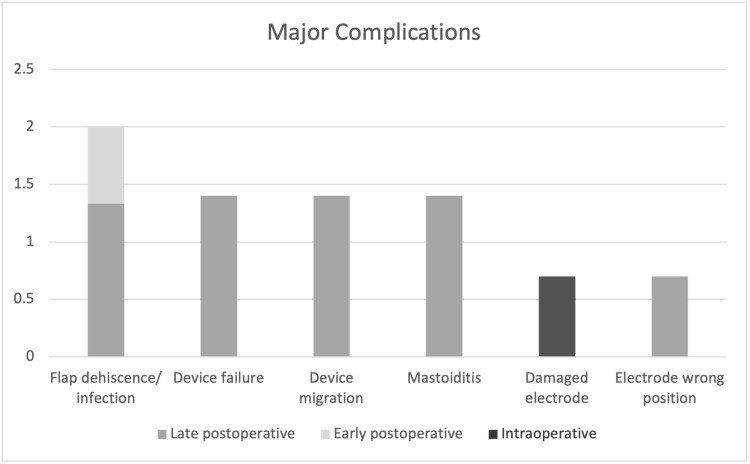
Percentage of complications according to time of appearance (Major complications, n=11)

Major complications occurred in 11 patients, including flap dehiscence/infection in three patients, device failure in two patients, device migration in two patients, mastoiditis in two patients, electrode damage during insertion in one patient, and misplaced electrodes in one patient (Table 3). Figure 1 and Figure 2 illustrate the degree of complications according to the time of appearance for minor and major complications.

## Discussion

The widespread dissemination of indications for cochlear implants and the effectiveness of this hearing rehabilitation method have significantly increased the number of implant patients worldwide [10]. However, complications related to surgery and equipment have been observed [11]. Therefore, surgical skills must be developed to avoid the complications associated with surgical techniques.

In recent years, the surgical procedure for implantation has changed to prevent surgical and medical complications. In addition, manufacturers of cochlear implants make every effort to prevent equipment malfunction and failure [10-12]. 

The complication rate of cochlear implants ranges between 3.7% and 24.6% in the literature [4,13-15]. Our study reported a minor complication rate of 11.5% and a major complication rate of 7.4%.

Minor complications

Cochleovestibular complications were noted to be the most frequent minor complications and were noted to be more prevalent in adults than in pediatric cases in our report. This can be attributed to the poor ability of children to express their complaints. Chen et al. reviewed 445 patients aged > 60 years who received CI between 1999 and 2011. The most common complications observed were balance problems in 30 patients (6.7%) [16].

Two adult patients reported early postoperative dizziness. This complication may be related to several factors such as loss of perilymph, aging of vestibular organs, and decreased blood flow. All patients were successfully treated with medication. Tinnitus was detected in two adult cases, and its intensity was worst in the first three months after implantations. 

Acute suppurative otitis media is common in children, however, it represents a serious complication of cochlear implant surgery such as implant extrusion, implant failure, or meningitis. In our report, three patients (2%) had acute suppurative otitis media that occurred late after surgery, all patients received empiric oral antibiotic treatment with complete improvement. Delayed complications of otitis media have also been reported by Loundon et al. in four among 434 patients; two patients were treated with antibiotics while the other two required ventilation tubes [17].

Postoperative facial nerve weakness is a rare complication. It can occur within two days due to edema or nerve injury, and it can be delayed after three days, for which few theories have been proposed. Reactivation of the herpes virus affecting facial nerve function is one of them, and it is well-documented after tympanomastoid procedures, including CI [18]. Moreover, surgical procedures with extensive nerve manipulation are associated with a high risk of viral reactivation [19]. In our study, transient facial nerve palsy was observed in 1.4% of patients. The patients were treated with prednisolone and antibiotics. One patient had a high possibility of an abnormal facial nerve course. A full resolution was observed in all patients within a few months. These findings are concordant with those reported by Chen et al. and Joseph et al. [16,20].

With minimal retroauricular incisions, two patients (1.4%) in our study had a wound infection. Both patients did not require further surgery and were treated with local and systemic antibiotics. Postoperative wound infections in relation to incision length have been investigated with the aid of Ray et al., who observed that implants that used larger incisions had a 2.4% prevalence of pores and surgical site infections. However, a smaller incision was associated with skin infections (1.1%) [21].

Malformation of the inner ear increases the complexity of the surgery and creates additional difficulties for the surgeon. Furthermore, an intraoperative gush of CSF upon cochlear opening can be seen in some cases. Previous studies reported a rate of intraoperative gushers between 0.4% and 5.58% [22-24], which is comparable to the rate found in this study (1.4%). To overcome these limitations, paying more attention to operative details can be of extraordinary assistance such as a small cochleostomy, conical external base arrays, and complete intraoperative sealing of the internal ear.

One of the common complications of CI is accidental facial nerve stimulation (FNS). The passage of electric current through the electrode to the spiral ganglion cell can spread to the nearby facial nerve, causing symptoms that range from simple awareness to severe facial spasm [25]. Cochlear disorders, such as otosclerosis, cochlear malformations, ossification, temporal bone fractures, and closed head injury, have been associated with a higher incidence of FNS. In the current study group, two patients with cochlear anomalies experienced facial nerve stimulation, one had an immediate onset of FNS and the other had a delayed onset. The increase in stimulation levels over time can explain the delayed onset of FNS. Both cases were managed with minimal changes in the speech processor fitting.

A delayed hematoma was observed in one patient who was treated with local wound care without surgical intervention. The delayed occurrence of symptoms was attributed to the children having thinner skin and bone tissue, leading to a tendency to be prone to soft-tissue trauma and subsequent infections. Similar findings were reported by Luiz et al. [26].

This study revealed that one patient (0.7%) suffered from chorda tympani injury (seen during the surgery without any postoperative manifestations). Postoperative taste dysfunction has been observed in 19.2% and 20% of patients, according to Al-Zahrani et al. [27] and Wagner et al. [28], respectively. The lower incidence found here could be due to the majority of our cases being children. 

Major complications

Infection is a major concern in CI surgery. The rate of complications related to infections ranges from 1.7% to 16.6% in the literature [29-30]. In our study, the most common major complication was skin flap infection. Two patients were managed with intravenous antibiotics and repeated wound dressings. Unfortunately, one patient had an infection refractory to medical treatment, which subsequently led to flap necrosis. Debridement and primary closure were performed for the treatment of partial necrosis and wound dehiscence. The use of smaller incisions, minimal hair shaving, and close observation of the skin overlying the magnet can reduce the rate of flap infection.

We registered in our series two cases of device failure (failures inducing permanent implant dysfunction) induced by trauma. This was confirmed by the dysfunction on the implant integrity test. Explantation was performed, followed by reimplantation during the same operation, and our data follow those presented in the literature [31].

Two patients (1.4%) experienced device migration. No symptoms were reported by either patient; however, the devices were at a lower than the normal position. In the second case, the device migrated antero-inferiorly and fell below the incision; therefore, the patient underwent revision surgery under general anesthesia. Repositioning of the device in the seat and fixation were performed. Black reported device migration in six out of 547 cases (1.1%), and the main cause was the creation of an overly large perichondrial pocket [32]. Methods to secure the device adequately include creating a smaller or tighter perichondrial pocket, a bony wall for stabilization, or suturing the device to the underlying bone.

There were two (1.4%) patients with late postoperative mastoiditis. Postauricular and intravenous antibiotics were administered to both patients. There were no intracranial problems associated with implant involvement; thus, surgery was not required.

The most crucial stage in cochlear implant surgery is the implantation of the cochlear implant electrodes. Since our center is a training facility, one electrode was damaged during insertion due to excessive manipulation of the electrode by a less experienced surgeon. The electrode was then replaced during the same session. In the report by Brito et al., the incidence of problems during electrode insertion was 3.8%. Among these, one bundle of electrodes was damaged during insertion [33].

Failure to identify anatomical landmarks during surgery, particularly the infracochlear air cell track or an undetected inner ear abnormality, could result in electrode array misplacement. Using intraoperative electrophysiological testing and postoperative imaging regularly should help prevent such complications. Electrode misplacement occurred in one patient in our study, despite normal intraoperative electrophysiological testing. Postoperatively, the patient was able to hear but could not discriminate between the sounds. Imaging confirmed that the electrode was located in the jugular bulb. Correction of the misplaced electrodes in the cochlea was confirmed using CT temporal bone postoperatively.

Bacterial meningitis, particularly pneumococcal meningitis, is more common in patients with cochlear implants. It must be emphasized that there was no incidence of postoperative meningitis in the KAUH Cochlear Implant Program, as the program includes vaccination against Streptococcus pneumoniae and Neisseria meningitides and administration of intravenous antibiotics.

## Conclusions

CI is a relatively safe and the most effective auditory rehabilitation method for patients with profound hearing loss with an acceptable complication profile. Our study indicated a minor complication rate of 11.5%, which resolved with simple medical intervention and a major complication rate of 7.5%. Four cases were related to device failure or migration and were managed surgically without further consequences. Electrode damage and misplacement were recorded in two cases and managed appropriately. Skin flap dehiscence occurred in three cases in which only one patient required debridement and primary closure due to flap necrosis, and two cases of mastoiditis were managed with antibiotics.

Appropriate indications, good preoperative imaging, consistent knowledge of the temporal bone anatomy, and meticulous surgical techniques could help decrease and prevent the complications related to CI surgery.
